# Characteristics and in vitro properties of potential probiotic strain *Fructobacillus tropaeoli* KKP 3032 isolated from orange juice

**DOI:** 10.1007/s12223-024-01207-7

**Published:** 2024-11-14

**Authors:** Anna Mikołajczuk-Szczyrba, Adrian Wojtczak, Marek Kieliszek, Barbara Sokołowska

**Affiliations:** 1 Department of Microbiology, Prof. Waclaw Dabrowski Institute of Agricultural and Food Biotechnology - State Research Institute, Rakowiecka 36 Street, Warsaw, 02-532 Masovian Voivodeship Poland; 2 Department of Food Biotechnology and Microbiology, Institute of Food Sciences, Warsaw University of Life Sciences–SGGW, Nowoursynowska 159C, 02-776 Warsaw, Poland

**Keywords:** FLAB, Fructophilic probiotic, Bile salts, Autoaggregation, Hydrophobicity, Antimicrobial activity

## Abstract

*Fructobacillus*, a Gram-positive, non-spore-forming, facultative anaerobic bacterium, belongs to the fructophilic lactic acid bacteria (FLAB) group. The group’s name originates from fructose, the favored carbon source for its members. *Fructobacillus* spp. are noteworthy for their distinctive traits, captivating the interest of scientists. However, there have been relatively few publications regarding the isolation and potential utilization of these microorganisms in the industry. In recent years, *F. tropaeoli* has garnered interest for its promising role in the food and pharmaceutical sectors, although the availability of isolates is rather limited. A more comprehensive understanding of *Fructobacillus* is imperative to evaluate their functionality in the industry, given their unique and exceptional properties. Our *in vitro* study on *Fructobacillus tropaeoli* KKP 3032 confirmed its fructophilic nature and high osmotolerance. This strain thrives in a 30% sugar concentration, shows resistance to low pH and bile salts, and exhibits robust autoaggregation. Additionally, it displays significant antimicrobial activity against foodborne pathogens. Evaluating its probiotic potential, it aligns with EFSA recommendations in antibiotic resistance, except for kanamycin, to which it is resistant. Further research is necessary, but preliminary analyses confirm the high probiotic potential of *F. tropaeoli* KKP 3032 and its ability to thrive in the presence of high concentrations of fructose. The results indicate that the isolate *F. tropaeoli* KKP 3032 could potentially be used in the future as a fructophilic probiotic, protective culture, and/or active ingredient in fructose-rich food.

**Fig. 1:**
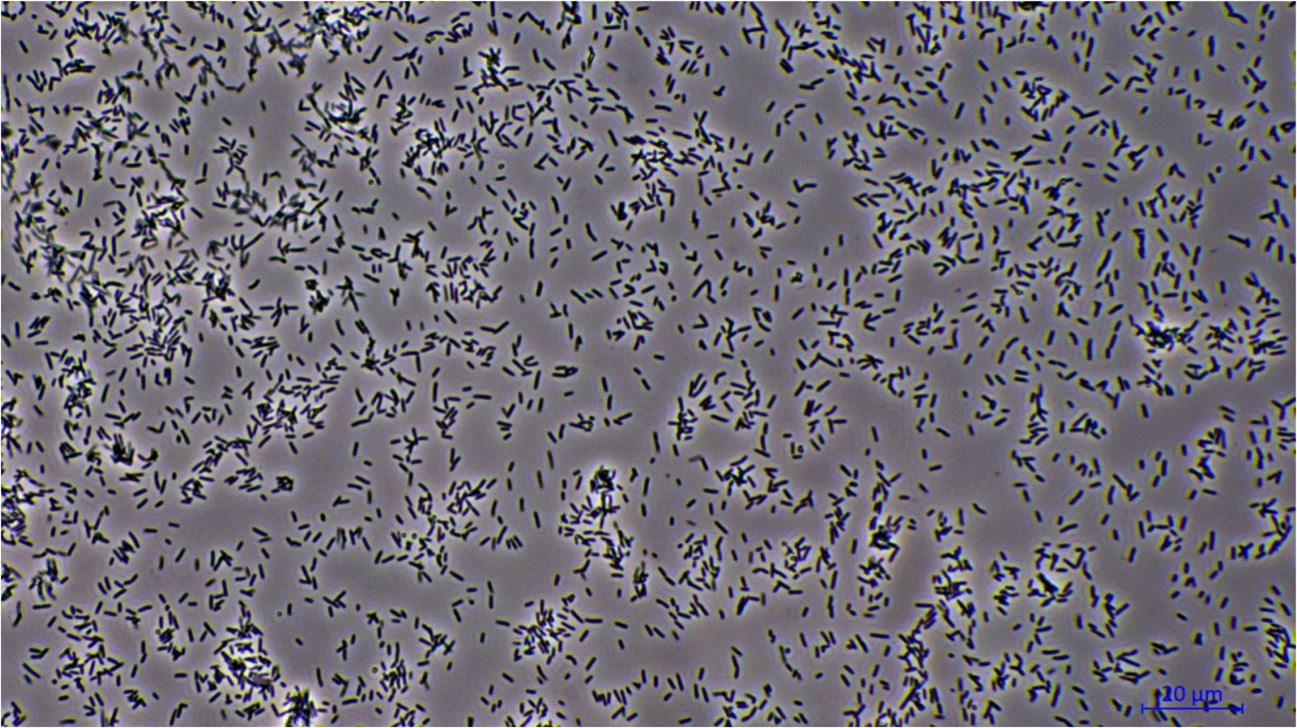
Microscopic view of *Fructobacillus tropaeoli* KKP 3032 at 600x magnification. The image showcases the distinct morphological features of the cells, which are characteristic of the *Fructobacillus* genus

## Introduction

Lactic acid bacteria (LAB) encompass a diverse group of microorganisms characterized by their common metabolic trait - the production of lactic acid. Recently, a novel category of bacteria has emerged within this group, referred to as FLAB (fructophilic lactic acid bacteria), comprising various species of *Fructobacillus* and *Lactobacillus*. The nomenclature of this group is rooted in fructose, which acts as the preferred carbon source for these microorganisms (Endo and Okada 2008; Endo et al. 2018). Initially, FLAB was classified as *Leuconostoc* spp.; nevertheless, through meticulous phylogenetic analysis and distinct biochemical properties, a separate genus, *Fructobacillus*, has been identified (Endo and Okada 2008).

At present, the genus *Fructobacillus* comprises eleven recognized bacterial species, including *F. fructosus, F. durionis, F. ficulneus, F. pseudoficulneus, F. tropaeoli, F. papyriferae, F. papyrifericola, F. broussonetiae, F. parabroussonetiae, F. cardui*, and *F. apis* (Mohamed et al. 2023). These microorganisms are primarily sourced from environments abundant in fructose, such as flowers, fruits, fruit-based fermented foods, and the digestive tracts of fructose-consuming insects, such as bees (Maeno et al. 2019). Species within the *Fructobacillus* genus are Gram-positive, non-spore-forming, facultatively anaerobic rods. When cultivated on agar plates, they typically yield diminutive, pale cream, and smooth colonies with diameters ranging from 1 to 2 mm. The optimal growth temperature for most of these bacteria is approximately 30$^\circ \textrm{C}$. Most *Fructobacillus* species possess highly efficient osmotic systems that facilitate growth in media containing 40% fructose or 5% NaCl (Endo and Okada 2008; Endo and Dicks 2014; Gustaw et al. 2017; Muslimah et al. 2021). Figure 1 presents a microscopic image of *Fructobacillus tropaeoli* KKP 3032, which is a representative species of the genus *Fructobacillus*.

Unlike LAB, FLAB demonstrates better growth on a medium enriched with D-fructose compared to D-glucose. Additionally, they necessitate the existence of an external electron acceptor in glucose metabolism. As noted by Endo et al. (2014, 2018), the fructophilic traits of FLAB stem from the absence of the *adhE* gene, which encodes a multifunctional protein responsible for alcohol dehydrogenase (ADH) and acetaldehyde dehydrogenase (ALDH) activities. These enzymes hold a pivotal role in the ethanol biosynthesis pathway and are indispensable for NAD^+^ regeneration in heterofermentative LAB bacteria. The deficiency in ADH and ALDH activity leads to a shortage of NAD^+^, thereby requiring the presence of electron acceptors in glucose metabolism. Furthermore, FLAB produces acetic acid from glucose instead of ethanol, a feature particularly distinguishing them from *Leuconostoc* (Gustaw et al. 2017).

FLAB, due to their distinctive properties, has garnered significant attention from the scientific community. Nevertheless, there is a scarcity of published studies that focus on their isolation and potential industrial applications (Sakandar et al. 2019). To date, only sporadic publications have addressed certain characteristics of FLAB, including their capacity for polyol production (Ruiz Rodríguez et al. 2017), their ability to break down complex compounds like lignin (Rokop et al. 2015), and even their intracellular synthesis of nicotinamide mononucleotide, a valuable industrial compound with anti-aging properties (Sugiyama et al. 2021). It has also been demonstrated that FLAB isolates could serve as potential candidates for grain fermentation in the baking industry. Notably, FLAB’s efficient utilization of fructose during grain fermentation may hold promise in mitigating irritable bowel syndrome (IBS), as fructose is recognized as a triggering factor for this condition (Sakandar et al. 2019). Moreover, there have been reports suggesting favorable effects of FLAB on the health of insects, particularly honeybees. However, their technological and probiotic potential in humans remains largely unexplored (De Simone et al. 2023; Pachla et al. 2021; Iorizzo et al. 2022; Zeid et al. 2022).

Currently, there remains a strong emphasis on the search for novel probiotic strains, despite the relatively extensive range of commercially available probiotics. The increasing demand for probiotics predominantly centers around microorganisms sourced from unconventional origins or possessing unique characteristics. These microorganisms hold the potential for the development of innovative and contemporary probiotic products or for enhancing the quality of existing ones (Sornplang and Piyadeatsoontorn 2016; Zielińska et al. 2015; Liu et al. 2022). Undoubtedly, FLAB bacteria, including *F. tropaeoli*, with their fructophilic characteristic being the most significant aspect, meet this growing demand. *Fructobacillus tropaeoli* was first isolated in South Africa from the *Tropaeolum majus* flower in 2009 by Endo et al. (2011), establishing it as a novel species within the *Fructobacillus* genus. In recent years, *F. tropaeoli* has garnered attention for its potential industrial applications, particularly in the realms of food and pharmaceuticals. However, the availability of isolates remains rather limited (Simsek et al. 2022). Moreover, the ability of *Fructobacillus tropaeoli* to thrive in fructose-rich environments suggests its utility as a fructophilic probiotic, protective culture, and/or active ingredient in fructose-containing food products such as jams, fruit juices, fruit preserves, jellies, thereby enhancing the nutritional value of these products.

In recent years, the utilization of fructose as a food ingredient has increased in significance due to its cost-effectiveness and relatively high sweetness perception compared to glucose and sucrose (Patil et al. 2020). Therefore, a fructophilic probiotic could be beneficial as a health-enhancing component, increasing the nutritional value of fructose-rich food products.

The objective of this study was to explore the potential of *F. tropaeoli* KKP 3032 as a potentially probiotic strain, aiming to expand our understanding of FLAB strains and their applications. By investigating its resilience under gastrointestinal conditions, ability to interact beneficially within the microbiome, and unique fructophilic properties, we seek to uncover new insights into its suitability for probiotic use. This research aims to contribute to the broader goal of identifying and utilizing FLAB strains in health and industry, paving the way for innovative probiotic formulations and technological applications.

## Materials and methods

### Identification

#### Molecular identification

The *Fructobacillus tropaeoli* KKP 3032 strain was obtained from the Culture Collection of Industrial Microorganisms-Microbiological Resources Center (IAFB). KKP 3032 was isolated from orange juice. The identification of the strain was confirmed by amplifying the 16 S rRNA gene region. Bacterial DNA was extracted using the commercial DNeasy PowerFood Microbial Kit from Qiagen GmbH, Hilden, Germany. Amplification was performed with the 16 S-F ($5'-AGAGTTTGATCCTGGCTCAG-3'$) and 16 S-R ($5'-ACGGCTACCTTGTTACGACTT-3'$) primers (Weisburg et al. 1991). PCR conditions for gene amplification were as follows: an initial denaturation at 95$^\circ \textrm{C}$ for 2 min, followed by 35 amplification cycles consisting of denaturation at 94$^\circ \textrm{C}$ for 30 s, hybridization at 51$^\circ \textrm{C}$ for 35 s, and extension at 72$^\circ \textrm{C}$ for 1 min. The process concluded with a final extension step at 72$^\circ \textrm{C}$ for 10 min, performed using the SimpliAmp™ Thermal Cycler from Applied Biosystems™  ThermoFisher Scientific, Waltham, MA, USA. The resulting amplicons were separated by electrophoresis on a 2% agarose gel containing the SimplySafe™ interfering compound (5 $\mu$L/100 mL; EURx, Gdansk, Poland). To estimate the size of the amplicons, a DNA Ladder with a size range of 100–3000 bp (A &A Biotechnology, Gdansk, Poland) was used. Electrophoresis was conducted at 110 V for 60 min using the Sub-Cell GT Horizontal Electrophoresis System from Bio-Rad, Madrid, Spain. The bands were visualized using the GeneFlash Network Bio Imaging System from Syngene, Wales, UK. Following this, sequencing was outsourced to the Genomed S.A. company in Warsaw, Poland. The raw sequences were analyzed using BLASTn from NCBI and deposited in the GenBank database.

#### MALDI-TOF MS identification

A 24-h culture of microorganisms grown on a solid medium underwent identification using MALDI-TOF MS (matrix-assisted laser desorption ionization time-of-flight mass spectrometry). In short, samples were analyzed using the Direct Smear plus Formic Acid (On-Target) method, employing the $\alpha$-cyano-4-hydroxy-cinnamic acid ($\alpha$-CHCA) matrix as per the manufacturer’s instructions. Mass spectrometry was performed using the MALDI-TOF MS instrument (AXIMA iD Plus Confidence, Shimadzu Corporation). *E. coli* DH5$\alpha$ served as the calibration standard for mass accuracy.

### Phylogenetic analysis

The phylogenetic analysis was conducted using MEGA 11 software. Molecular sequences obtained for the specified genes were aligned utilizing ClustalW through the “Align” option in MEGA 11. The evolutionary model, specifically the Maximum Composite Likelihood model, was selected to represent the underlying genetic changes. The number of bootstrap replications employed for branch reliability assessment was set to 10,000, providing robust statistical support for the constructed phylogenetic tree. The Neighbor-Joining Tree method was then applied to generate the final phylogenetic tree, visualizing the evolutionary relationships among the analyzed sequences. The resulting tree was exported for further analysis (Gustaw et al. 2018).

### Biochemical characterization

The analysis of carbohydrate fermentation was conducted using the API CHL 50 kit (BioMérieux, Marcy l´Etoile, France) following the manufacturer’s instructions. In summary, the inoculum, prepared in API Suspension Medium ampoules (equivalent to a turbidity of 2 on the McFarland scale using a densitometer DEN-1B, Biosan, Poland), was added to the wells. The wells were then covered with mineral oil and incubated at a temperature of 37$^\circ \textrm{C}$C for a period ranging from 24 to 96 h, with daily observations. The observations were based on changes in color within the wells, and the results were interpreted according to the instructions provided with the kit (Simsek et al. 2022). Catalase activity was determined by subjecting it to a reaction with 3% (v/v) H_2_O_2_ on FYP agar (30$^\circ \textrm{C}$, 48 h).

### Fructophilic properties

To assess the fructophilic properties, we employed the Bioscreen C Pro automated growth curve analysis system (Oy AB Ltd., Growth Curves, Finland). The growth kinetics of the *F. tropaeoli* KKP 3032 strain were estimated using the methodology outlined by Kiousi et al. (2023). The study utilized the following media: FYP (fructose yeast peptone) broth (10 g/L D-fructose, 10 g/L yeast extract, 5 g/L polypeptone, 2 g/L sodium acetate, 0.5 g/L Tween 80, 0.2 g/L MgSO$_4\cdot$7 H$_2$O, 0.01 g/L MnSO$_4\cdot$4 H$_2$O, 0.01 g/L FeSO$_4\cdot$7 H$_2$O, 0.01 g/L NaCl; pH 6.8) (Endo et al. 2011), along with modified versions: GYP (glucose yeast peptone) broth (10 g/L D-glucose instead of D-fructose), and GYP-F (5 g/L D-glucose and 5 g/L D-fructose), both representing variations with a different type of sugar while otherwise maintaining the same composition as FYP.

Before the experiment, a cultivation period of 18 h at 30$^\circ \textrm{C}$ on FYP broth was conducted. Subsequently, the culture was adjusted to OD of 0.5. Following this, 50 $\mu$L of a 0.5 McF microbial culture (equivalent to $10^7$ CFU/mL) was inoculated into FYP broth and applied to wells containing 250 $\mu$L of medium. The research was carried out for 24 h at 30$^\circ \textrm{C}$, with OD$_{600}$ measurements taken every hour from each of the five wells. Simultaneously, anaerobic cultivation was performed, with all procedures identical except that the wells were additionally covered with paraffin.

As negative controls, non-inoculated FYP, GYP, and GYP-F were employed.

The experiment was replicated five times, with each sample placed in five different wells. After obtaining the growth curves, Gompertz curves were generated.

In order to assess differences between groups, a one-way analysis of variance (ANOVA) was utilized.

Moreover, to explore the impact of oxygen on bacterial growth, the strains were inoculated onto GYP agar with D-glucose as the exclusive carbon source and incubated under both anaerobic and aerobic conditions at 30$^\circ \textrm{C}$ for 48 h. Anaerobic conditions were maintained using a gas generation kit (GENbag anaer, BioMérieux, France). This investigation was carried out for both *Fructobacillus tropaeoli* KKP 3032 and *Leuconostoc mesenteroides* KKP 389.

### Sugar tolerance

To evaluate sugar tolerance, we employed the automated growth curve analysis system Bioscreen C Pro (Oy AB Ltd., Growth Curves, Finland) to estimate the growth kinetics of the *F. tropaeoli* KKP 3032 strain, following the method outlined by Kiousi et al. (2023). In the course of the study, solutions with varying fructose concentrations were meticulously prepared using FYP medium as the foundational base. These solutions were labeled as FYP10 (10% fructose), FYP20 (20% fructose), FYP30 (30% fructose), and FYP40 (40% fructose). Simultaneously, the control group consisted of FYP medium with a 1% fructose concentration.

Prior to the experiment, a cultivation period of 18 h was conducted at a temperature of 30$^\circ \textrm{C}$ on FYP agar. Following this, the culture was adjusted to an absorbance (A) of 0.5. Subsequently, 50 $\mu$L of a 0.5 McF microbial culture (equivalent to $10^7$ CFU/mL) was inoculated in FYP broth and applied to wells containing 250 $\mu$L medium. The research was carried out for 24 h at 30$^\circ \textrm{C}$, and A$_{600}$ measurements taken every hour from each of the wells.

As negative controls, non-inoculated FYP and all media with fructose concentrations were utilized.

The experiment was replicated five times, with each sample placed in five different wells. After obtaining the growth curves, Gompertz curves were generated.

In order to assess differences between groups, a one-way analysis of variance (ANOVA) was utilized.

### Growth characteristics of microorganisms

After obtaining the growth curves, Gompertz curves were generated following the methodology outlined by Kiousi et al. (2023) using the LabPlot 2.9.0 program (KDE).

1 $$\begin{aligned} L_t = A + C \cdot e^{-e^{-B \cdot (t - D)}} \end{aligned}$$

where

*Lt*: A at time *t*
*t*: time (h)
*A*: asymptotic A value as *t* decreases indefinitely
*B*: relative growth rate at *D*
*C*: the asymptotic amount of growth that occurs as *t* increases indefinitely
*D*: time at which the absolute growth rate is at its maximum (h)

The maximum growth rate $\mu _{max}$ was determined based on the Gompertz model.

2 $$\begin{aligned} \mu _{max} = \frac{B \times C}{e} \times h^{-1} \end{aligned}$$

The change in absorbance ($\Delta A$) was determined based on the difference between $A_{max}$ and $A_{min}$:

3 $$\begin{aligned} \Delta A = A_{max} - {A}_{min} \end{aligned}$$

where

$$A_{max}$$:
the highest value of absorbance observed during the process;
$$A_{min}$$:
the lowest value of absorbance observed during the process.

In order to assess differences between groups, a one-way analysis of variance (ANOVA) was utilized.

### Probiotic potential

#### Tolerance against low pH

Tolerance against low pH was assessed using a modified method according to Zielińska et al. (2015). The *Fructobacillus tropaeoli* KKP 3032 strain was cultured for 24 h in Fructose de Man, Rogosa, and Sharpe agar (FMRS) broth (MRS; with 10 g/L D-fructose). Afterward, it was centrifuged, washed twice with phosphate-buffered saline (PBS), and resuspended in PBS buffer ($pH=7.2$) to achieve an absorbance of 0.5 McFarland units. Subsequently, 1 mL of the microbial suspension was transferred into 9 mL of fresh PBS buffer at $pH$ 1.5, 2.0, and 3.0, and incubated for 2 h at $37^\circ C$ to investigate the pH effect. Microbiological cultures were plated after 30 and 120 min of incubation. Following each time point, the bacteria were spread in 100 $\mu {l}$ submultiples on FMRS agar medium and incubated for 48 h at $30^\circ C$ to assess their growth. The survival of *Fructobacillus tropaeoli* KKP 3032 (in %) was calculated as follows (Zielińska et al. 2015):

4 $$\begin{aligned} Survival (\%) = \frac{N_i}{N_x} \times 100 \end{aligned}$$

where

$$N_i$$:
log CFU/mL after incubation,
$$N_x$$:
log CFU/mL before incubation

In order to assess differences between groups, a one-way analysis of variance (ANOVA) was utilized.

#### Bile salt tolerance

Bile salt tolerance was assessed using a modified method according to Zielińska et al. (2015). The *F. tropaeoli* KKP 3032 strain was cultured for 24 h in FMRS broth. Subsequently, it was centrifuged, subjected to two washes with phosphate-buffered saline (PBS), and then resuspended in PBS buffer ($pH=7.2$) to attain an absorbance of 0.5 McFarland units. Following this, 1 mL of the microbial suspension was transferred into 9 mL of fresh FMRS broth, with the addition of 0.3%, 0.5%, 0.7%, and 1.5% (w/v) bile salts (Ox bile powder; Sigma Aldrich). The impact of bile salts was evaluated after 24 and 48 h of incubation at $37^\circ C$. The bacteria were subsequently inoculated (100 $\mu {l}$ aliquots) onto FMRS agar medium and incubated for 48 h at $30^\circ C$. The survival rate of *F. tropaeoli* KKP 3032 (expressed as a percentage) was computed using the following formula (Zielińska et al. 2015):

5 $$\begin{aligned} Survival (\%) = \frac{N_i}{N_x} \times 100 \end{aligned}$$

where

$$N_i$$:
log CFU/mL after incubation,
$$N_x$$:
log CFU/mL before incubation

In order to assess differences between groups, a one-way analysis of variance (ANOVA) was utilized.

#### Autoaggregation test

The *Fructobacillus tropaeoli* KKP 3032 strain was cultured in FMRS broth for 24 h at $30^\circ C$. Cell pellets were obtained by centrifugation at $5000 \, g$ and $4^\circ C$ for 15 min, followed by three washes with PBS at $pH \, 7.2$, adjusted to a absorbance corresponding to McFarland 0.5 standard. A $5 \, mL$ suspension was vortexed for $10$ s and then incubated at $37^\circ C$. After $6$ h, a subset ($n=3$) of samples was measured for absorbancey (A) at $600 \, nm$, while the remaining samples ($n=3$) were further incubated for a total duration of $24$ h before measurement. After this incubation period, absorbance (A) was measured at $600 \, nm$ to assess auto-aggregation. The auto-aggregation percentage was calculated as

6 $$\begin{aligned} Autoaggregation (\%) = \frac{A_{t0} - A_{tx}}{A_{t0}} \times 100 \end{aligned}$$

where

$$A_{t0}$$
is absorbance at the beginning,
$$A_{tx}$$
is absorbance after incubation time.

The experiment was replicated three times (Simsek et al. 2022).

#### Antimicrobial activity

Antimicrobial activity was assessed using the agar well diffusion assay. Various food-borne pathogenic bacteria, including *Listeria monocytogenes* KKP 1058, *Pseudomonas aeruginosa* KKP 994, *Staphylococcus aureus* KKP 995, *Salmonella enterica* KKP 1044, *Escherichia coli* KKP 987, and *Bacillus cereus* KKP 358, were cultured on nutrient agar (Merck, Germany) at 37$^\circ \textrm{C}$ for 24 h. A microbial suspension with a density of approximately $10^7$ CFU/mL for each pathogen was prepared in saline. *Fructobacillus tropaeoli* KKP 3032 was cultivated in FMRS broth at 30$^\circ \textrm{C}$ for 24 h. Cell-free culture supernatants (CFCSs) were obtained by centrifuging the FMRS broth at 10,000 g for 10 min. Next, pathogenic bacteria were sub-cultured on nutrient agar, and 100 $\mu _{L}$ of the CFCSs were placed into wells on the nutrient agar plates, which were then incubated at 37$^\circ \textrm{C}$ for 24 h. The diameter of the inhibition zones around the wells was measured. Isolates with clear inhibition zones measuring less than 11 mm, between 11 and 16 mm, between 17 and 22 mm, and more than 23 mm were classified as negative (-), mild (+), strong (++), and very strong (+++) inhibitors, respectively. Sterile FMRS broth was used as a negative control (Lashani et al. 2020).

#### Bacterial adhesion to hydrocarbons

The bacterial adhesion to hydrocarbons (BATH) test was conducted following the procedure outlined by Reniero et al. (1992). Bacterial cells, obtained through a 24-h cultivation in FMRS broth at 30$^\circ \textrm{C}$, were thoroughly washed with a PBS buffer (pH=7.2) and subsequently suspended in the same buffer. The absorbance was adjusted to $0.25 \pm 0.05$ at 600 nm using a Genesys 10 S UV–Vis spectrophotometer (Thermo, Waltham, MA USA) to standardize the bacterial count to $10^8$ CFU/mL. Subsequently, equal volumes of the viable bacterial suspension and the solvent (xylene, POCH, Gliwice, Poland) were thoroughly mixed by vortexing for 5 min. After 1 h of incubation at room temperature, the aqueous phase was carefully removed, and its absorbance was measured. The results were expressed as percentages using the following formula:

7 $$\begin{aligned} BATH \% = \left( \frac{A_0 - A}{A_0} \right) \times 100 \end{aligned}$$

where $A_0$ and $A$ are absorbance before and after mixing with xylene, respectively (Reniero et al. 1992).

**Fig. 2:**
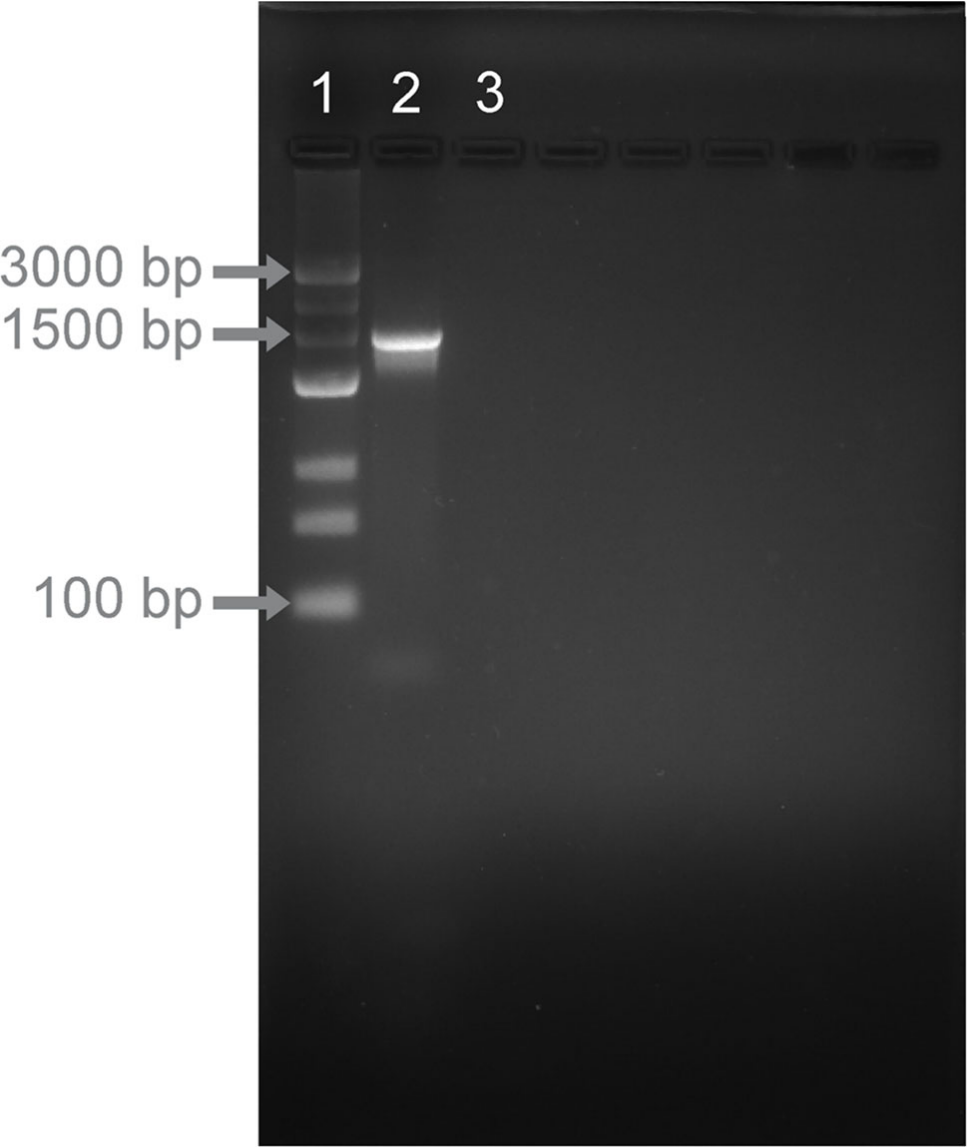
Agarose gel electrophoresis of the 16 S rRNA gene amplicons of the KKP 3032 strain. Lanes: 1 = ladder; 2 = KKP 3032; 3 = negative control

#### Safety evaluation — antibiotic susceptibility

The antibiotic resistance profile of the *Fructobacillus tropaeoli* KKP 3032 strain was assessed using the E test (bioMérieux, France) on FMRS agar according to the manufacturer’s instructions. The strain was classified as resistant if their MIC values exceeded the MIC breakpoints established by the EFSA (Zielińska et al. 2015; Rychen et al. 2018).

**Fig. 3:**
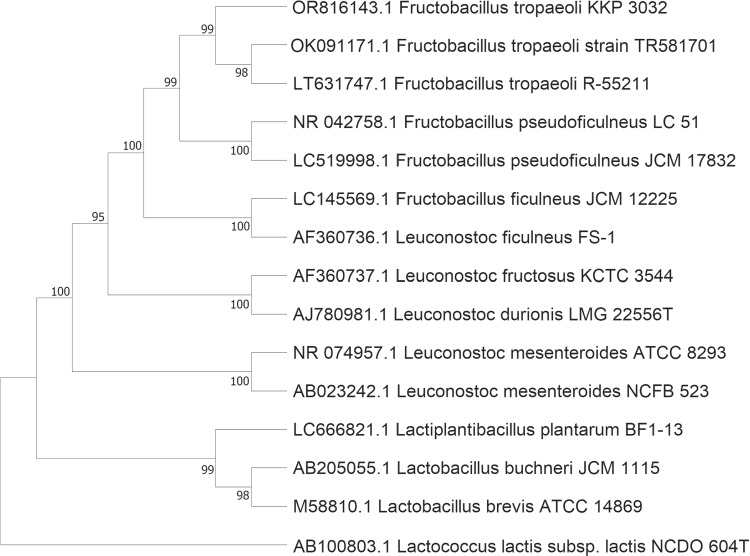
Phylogenetic relationships between *Fructobacillus tropaeoli* KKP 3032 and related species based on 16 S rRNA gene sequences. The tree was constructed by the neighbour-joining method. *Lactococcus lactis* subsp. *lactis* NCDO 604T was used as an outgroup

### Statistical analysis

The statistical analyses were conducted using a one-way analysis of variance (ANOVA). Tukey’s HSD test ($\alpha =0.05$) was subsequently employed to assess pairwise differences. The results are presented as homogeneous groups on a graph. The normality distribution was checked using the Shapiro-Wilk test. The statistical software used for these analyses was Statistica 14.0 (TIBCO Software, Palo Alto, CA, USA).

## Results and discussion

### Identification

#### Molecular identification

The KKP 3032 strain was isolated from spoiled commercial orange juice. The identification of the isolate KKP 3032 was carried out by analyzing sequences of the 16 S rRNA gene. The gel image is shown in Fig. 2. Nucleotide BLAST analysis revealed that the KKP 3032 isolate belongs to the phylum *Firmicutes* and the family *Bacillaceae*. The strain exhibited a 99.85% similarity in the nucleotide sequence of the 16 S rDNA gene to the identified *Fructobacillus tropaeoli*.

The phylogenetic analysis revealed significant similarity, with the closest resemblance observed to *Fructobacillus tropaeoli*. This finding underscores the evolutionary proximity of the analyzed sequences to *Fructobacillus tropaeoli*, suggesting a shared genetic heritage and potential common ancestry (see Fig. 3).

The strain has been deposited in the Culture Collection of Industrial Microorganisms - Microbiological Resources Center (IAFB) as *Fructobacillus tropaeoli* KKP 3032 and is also cataloged in the NCBI Gene Bank under accession number OR816143.

#### MALDI-TOF MS identification

Additionally, besides identification based on 16 S rRNA sequencing, an analysis was conducted using MALDI-TOF MS. This method involves analyzing the “fingerprint” of ribosomal proteins characteristic of a particular family, genus, or species. MALDI-TOF MS enables the identification of mass spectral profiles of the tested strains by comparing them with a library containing reference spectra. In contrast to genomic techniques, where analyzed sequences are compared in a continuously expanding and updated global database like NCBI BLAST (Basic Local Alignment Search Tool), in the MALDI-TOF MS technique, the identification of microorganisms is carried out using internal databases specific to the particular instrument (Bucka-Kolendo and Sokołowska 2021). However, these databases do not contain protein profiles of all bacterial species, which is why this method has certain limitations and mainly depends on the library resources of the specific instrument.

**Fig. 4:**
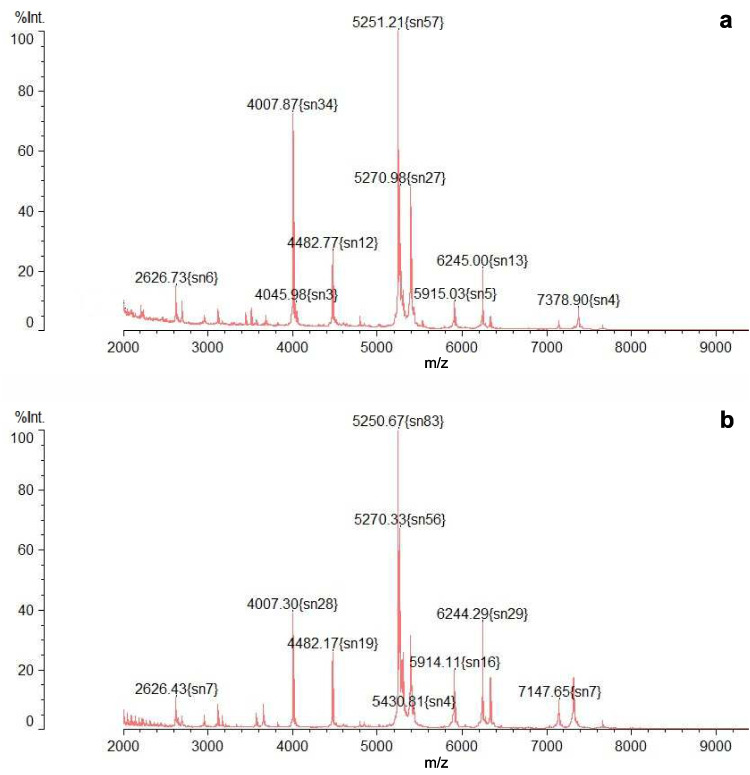
Comparison of mass spectra of *F. tropaeoli* strains DSM 23246 (**a**) and KKP 3032 (**b**) obtained using MALDI-TOF MS method

*F. tropaeoli* is a rare and relatively unknown species, with its protein profile absent from our database. Consequently, due to the lack of a spectrum standard in the MALDI-TOF MS database, a comparison was made between the protein profile of strain *F. tropaeoli* KKP 3032 and that of the reference strain *F. tropaeoli* DSM 23246. The obtained protein profiles of the tested strains are depicted in Fig. 4. The protein spectra of the tested strains exhibited striking similarities, suggesting a close relationship between these strains. Specifically, the tested strains shared 7 common peaks (some with varying intensities), with peaks observed at approximately 2626, 4007, 4482, 5251, 5270, 5915, and 6245 m/z. Additionally, the spectrum of strain *F. tropaeoli* KKP 3032 featured peaks at 5430 and 7147 m/z, which were absent in the reference strain *F. tropaeoli* DSM 23246. Conversely, examination of the spectrum of strain *F. tropaeoli* DSM 23246 revealed the presence of two peaks at 4045 and 7378 m/z, which were not detected in *F. tropaeoli* KKP 3032. These slight differences in protein profiles are most likely due to strain-level differences. Furthermore, the occurrence of minor differences in protein spectra is evident due to the development of different strategies enabling the survival of microorganisms under stressful conditions (UV light and/or oxidative damage). As a result of the conducted analyses, it was found that the use of this non-standard method is a valuable complement to other identification methods.

### Biochemical characterization

In the initial phase of the research, the biochemical properties of the strain were determined using the API CHL 50 kit. The *Fructobacillus tropaeoli* KKP 3032 strain fermented only four out of the total forty-nine carbohydrates (Table 1). The obtained carbohydrate fermentation profile aligns with prior studies, which indicated that FLAB group bacteria typically metabolize a limited number of carbohydrates, usually ranging from 2 to 4. This consistency in carbohydrate utilization patterns underscores the characteristic metabolic traits of FLAB bacteria, providing additional support for the broader understanding of their carbohydrate fermentation capabilities (Meradji et al. 2023; Endo et al. 2009). Fructose was the carbohydrate fermented most rapidly, with a noticeable change in well-color observed on the first day. This feature has only been reported in the case of FLAB and has not been observed in any other LAB. Therefore, it appears to be one of the fundamental, shared characteristics of all fructophilic LAB (Endo and Okada 2008; Endo et al. 2009). On the second and third days, glucose and mannitol fermentation were recorded, respectively. Interestingly, on the fourth day, it was observed that the KKP 3032 strain could metabolize potassium gluconate, which had not been previously noted in the *Fructobacillus tropaeoli* metabolism profile (Endo et al. 2011). Conversely, other researchers noted that the production of acid from D-galactose, mannitol, melibiose, trehalose, and potassium gluconate is a strain-specific trait (Meradji et al. 2023; Endo et al. 2012). Further research is needed to clarify this matter.

**Table 1:** Carbohydrates fermented by tested strains detected through API 50 CHL kit

	Fermented carbohydrates			
	Glucose	Fructose	Mannitol	Potassium gluconate
*Fructobacillus tropaeoli*	Day 2	Day 1	Day 3	Day 3^w^
KKP 3032				

^w^ - weak growth

Acid production was not observed when glycerin (glycerol), erythritol, D-ribose, D- and L-arabinose, D- and L-xylose, D-ribitol (D-adonitol), methyl-$\beta$-D-xyloside, D-galactose, D-mannose, L-sorbose, L-rhamnose, myo-Inositol, D-sorbitol (D-Glucitol), methyl-$\alpha$-D-mannopyranoside, D-galactitol (dulcitol), methyl-$\alpha$-D-glucopyranoside, amygdalin, N-Acetyl-D-glucosamine, arbutin, esculin, salicin, D-maltose, $\beta$-lactose (D-lactose), D-sucrose, D-trehalose, inulin, D-melibiose, starch, D-cellobiose, D-melezitose, glycogen, xylitol, D-raffinose, gentiobiose, D-turanose, D-lyxose, D-tagatose, potassium 2-ketogluconate, potassium 5-ketogluconate D- and L-fucose, D-arabinitol (D- and L-arabitol), were used as substrates.

Under aerobic conditions and in FYP agar (30$^\circ \textrm{C}$, 48 h), the *Fructobacillus tropaeoli* KKP 3032 strain tested negative for catalase activity.

**Table 2:** Results for growth rate coefficients ($\mu$) andabsorbance difference ($\Delta$A) for *Fructobacillus tropaeoli* KKP 3032 strain (*n* = 5)

Medium	$\mu _{max}$	$\Delta$A
OXY-FYP	$0.154 \pm 0.003$^b^	$0.878 \pm 0.015$^d^
ANA-FYP	$0.144 \pm 0.004$^b^	$0.823 \pm 0.013$^c^
OXY-GYP	$0.080 \pm 0.004$^a^	$0.647 \pm 0.021$^b^
ANA-GYP	$0.074 \pm 0.001$^a^	$0.354 \pm 0.022$^a^
OXY-GYP-F	$0.285 \pm 0.001$^c^	$0.964 \pm 0.011$^e^
ANA-GYP-F	$0.301 \pm 0.006$^c^	$0.834 \pm 0.011$^c^

**Note:** Lowercase letters indicate statistically significant differences for $\mu _{max}$ and $\Delta$A between medium variants

### Growth characteristics — fructophilic properties

**Fig. 5:**
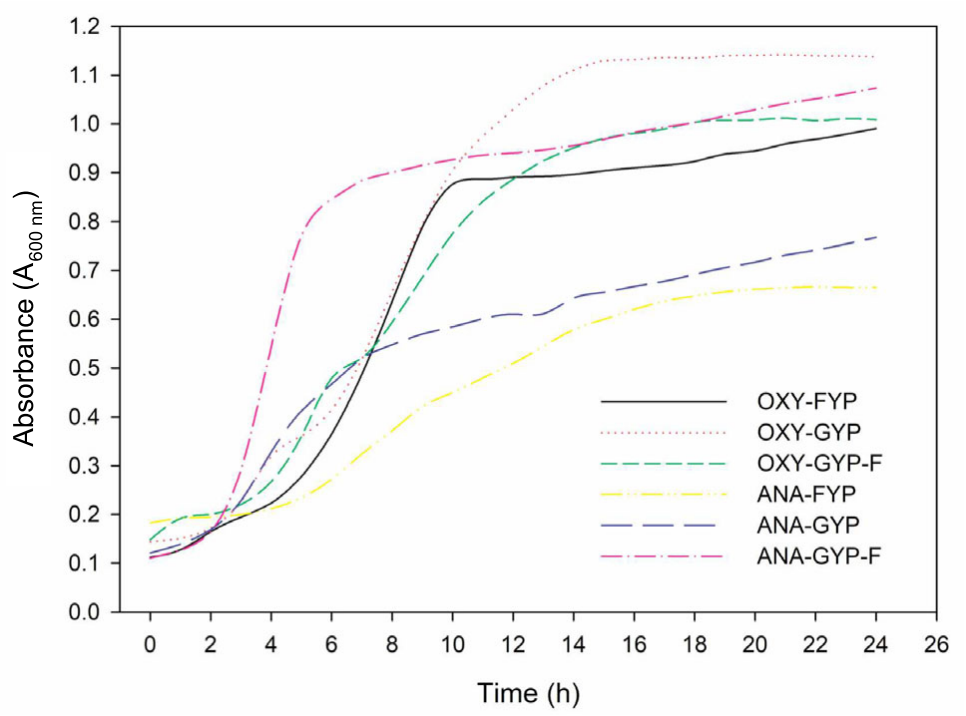
Growth curves of the strain *Fructobacillus tropaeoli* KKP 3032 in aerobic (OXY) and anaerobic (ANA) conditions on media containing glucose (GYP), fructose (FYP), or their mixture (GYP-F) (*n* = 5)

The provided results offer insights into the growth dynamics of microorganisms across various culture conditions, focusing on the parameters $\mu _{max}$ (relative growth rate) and $\Delta$A (absorbance change) (Table 2, Fig. 5). In terms of $\mu _{max}$, the aerobic condition with fructose (OXY-FYP) and its anaerobic counterpart (ANA-FYP) both demonstrated relatively similar growth rates, around 0.154 and 0.144, respectively. However, the aerobic glucose condition (OXY-GYP) displayed a lower growth rate of 0.080, while the anaerobic glucose condition (ANA-GYP) showed an even lower rate of 0.074. Intriguingly, media with a combination of fructose and glucose (OXY-GYP-F and ANA-GYP-F) exhibited the highest growth rates, with values of 0.285 and 0.301, respectively.

Turning to the $\Delta$A values, which reflect changes in absorbance and, consequently, cell density, OXY-FYP and ANA-FYP while seemingly similar, actually exhibited statistically significant differences, with $\Delta$A values of 0.878 and 0.823, respectively. Under aerobic conditions, OXY-GYP displayed a significantly higher $\Delta$A of 0.647 compared to the anaerobic condition ANA-GYP, which had a $\Delta$A of 0.354. Notably, the combination of fructose and glucose in both aerobic and anaerobic conditions (OXY-GYP-F and ANA-GYP-F) resulted in the highest $\Delta$A values, indicating substantial growth, with values of 0.964 and 0.834, respectively. Cell density on ANA-FYP and ANA-GYP-F did not differ significantly.

These findings lead to several conclusions. Firstly, media containing fructose (FYP) generally support higher growth rates ($\mu _{max}$) compared to glucose-containing media (GYP) under both aerobic and anaerobic conditions. Secondly, among aerobic conditions, the media with a combination of fructose and glucose (OXY-GYP-F) exhibit the highest growth rate. Thirdly, anaerobic growth in general (ANA) appears to result in lower growth rates compared to aerobic conditions. Lastly, the addition of both fructose and glucose in the media (GYP-F) seems to enhance growth compared to media with only glucose (GYP) under aerobic conditions (Table 2, Fig. 5).

**Fig. 6:**
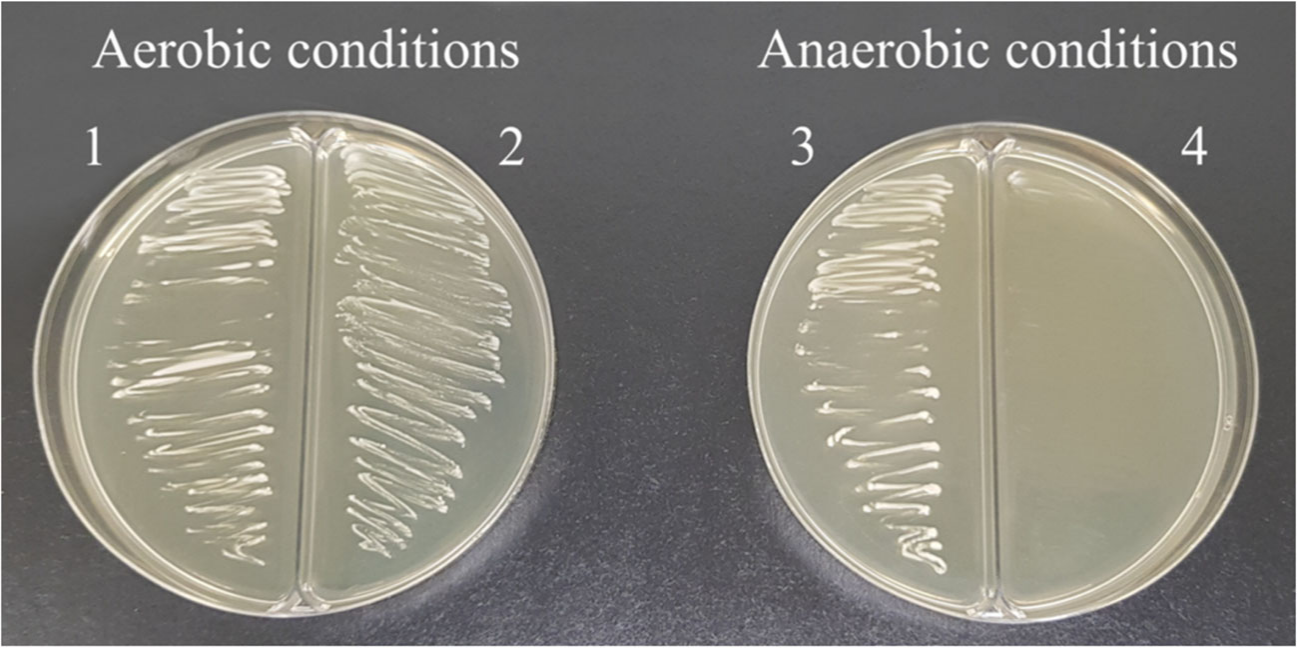
Growth of *L. mesenteroides* KKP 389 and *F. tropaeoli* KKP 3032 on GYP agar under aerobic (left) and anaerobic (right) conditions. 1,3 - *L. mesenteroides* KKP 389; 2,4 - *F. tropaeoli* KKP 3032

Additionally, the results suggest that the *F. tropaeoli* KKP 3032 strain can be classified as fructophilic, as it exhibits weaker growth on the GYP medium under anaerobic conditions. This observation implies that the strain might rely on fructose as a preferred carbon source, and its growth is compromised in the absence of an external electron acceptor in the form of oxygen. The diminished growth on GYP under anaerobic conditions further supports the notion that the *F. tropaeoli* KKP 3032 strain is adapted to environments where fructose is readily available, and its growth benefits from aerobic conditions with an electron acceptor.

According to Endo et al. (2009), characteristics of a fructophilic strain include, among others: (1) a preference for D-fructose over D-glucose, (2) a requirement for electron acceptors for the dissimilation of D-glucose, (3) utilization of oxygen as an electron acceptor for growth, and (4) a preference for aerobic rather than anaerobic conditions due to limited sugar fermentation capabilities.

In conclusion, the observed growth patterns of the *F. tropaeoli* KKP 3032 strain, particularly its preference for fructose and reliance on aerobic conditions with an electron acceptor, align with the criteria outlined by Endo et al. (2009) for fructophilic strains. This study provides empirical evidence supporting the classification of the *F. tropaeoli* KKP 3032 strain as fructophilic, contributing to a more comprehensive understanding of its metabolic characteristics and ecological niche preferences.

The plate test conducted to assess the strain *Fructobacillus tropaeoli* KKP 3032’s response to oxygen revealed robust growth under aerobic conditions. However, under anaerobic conditions on GYP medium, the strain exhibited notably limited growth. These findings imply that the KKP 3032 strain relies on an external electron acceptor during glucose metabolism, with oxygen efficiently serving in this capacity. Interestingly, the presence or absence of oxygen did not seem to influence the growth of *Leuconostoc* mesenteroides KKP 389 (Fig. 6).

Our findings are consistent with those of Endo et al. (2018), confirming the necessity of an external electron acceptor in the metabolism of glucose by *Fructobacillus* spp. According to current knowledge, this requirement is likely attributed to the complete or partial deletion of the *adhE* gene, which encodes the bifunctional alcohol-aldehyde dehydrogenase, AdhE. The AdhE enzyme plays a pivotal role in the phosphoketolase pathway and is essential for maintaining the NAD+/NADH balance during the metabolism of D-glucose in heterofermentative LAB. In the case of FLAB, external electron acceptors oxidize NADH to NAD+ and sustain the NAD+/NADH equilibrium (Endo et al. 2018; Maeno et al. 2021). In support of this hypothesis, research conducted by Maeno et al. (2019). demonstrated that the introduction of the *adhE* gene into FLAB significantly modified their metabolic characteristics, enabling them to metabolize D-glucose in the absence of electron acceptors. Species within the *Fructobacillus* genus are typically isolated from environments abundant in fructose, rendering glucose metabolism unnecessary for them. The metabolism of fructose in heterofermentative LAB proceeds without the involvement of the *adhE* gene, as fructose, serves as both a carbohydrate source and an electron acceptor. This suggests that *Fructobacillus* spp. may have lost the *adhE* gene during their adaptation to fructoserich habitats (Endo et al. 2014).

### Sugar tolerance

**Fig. 7:**
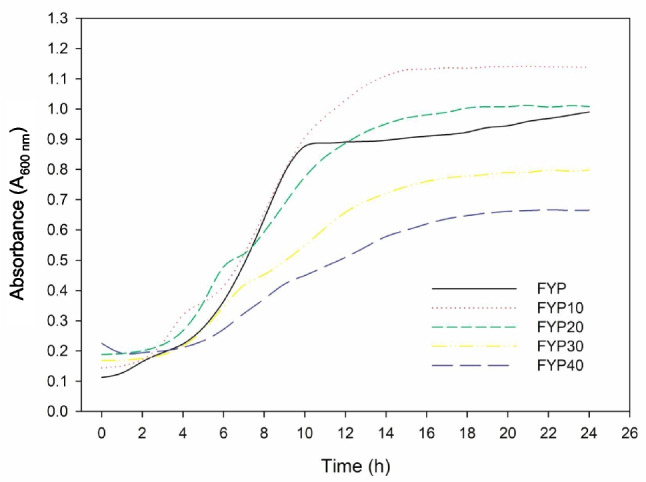
Growth curves of the strain *Fructobacillus tropaeoli* KKP 3032 in FYP media with varying fructose concentrations (*n* = 5). FYP contains 1% fructose, while FYP10, FYP20, FYP30, and FYP40 contain 10%, 20%, 30%, and 40% fructose, respectively

The growth characteristics of the *F. tropaeoli* KKP 3032 strain were investigated under varying fructose concentrations, focusing on $\mu _{max}$ and $\Delta$A (Table 3, Fig. 7).

**Table 3:** Results for growth rate coefficients ($\mu$) and absorbance difference ($\Delta$A) for *Fructobacillus tropaeoli* KKP 3032 strain (*n* = 5)

Medium	$\mu _{max}$	$\Delta$A
FYP	$0.154 \pm 0.003$^e^	$0.878 \pm 0.015$^e^
FYP10	$0.127 \pm 0.004$^d^	$0.994 \pm 0.013$^d^
FYP20	$0.112 \pm 0.001$^c^	$0.820 \pm 0.011$^c^
FYP30	$0.063 \pm 0.007$^b^	$0.629 \pm 0.011$^b^
FYP40	$0.042 \pm 0.003$^a^	$0.441 \pm 0.056$^a^

**Note:** Lowercase letters indicate statistically significant differences for $\mu _{max}$ and $\Delta$A between medium variants

For $\mu _{max}$, the highest growth rate was observed in the aerobic condition with 1% fructose (FYP), reaching 0.154. As the fructose concentration increased, a gradual decline in growth rates was noted: 0.127 for FYP10 (10% Fructose), 0.112 for FYP20 (20% Fructose), 0.063 for FYP30 (30% Fructose), and 0.042 for FYP40 (40% Fructose). This decreasing trend suggests that the strain’s growth is influenced by the concentration of fructose in the medium.

Examining $\Delta$A values, it is noteworthy that FYP10 exhibited a notable increase in cell density, surpassing even FYP. Specifically, FYP10 demonstrated a $\Delta$A value of 0.994, indicating higher growth or cell density under the condition of 10% fructose compared to the 1% fructose medium (FYP), which had a $\Delta$A value of 0.878. In contrast, as fructose concentration increased further in FYP20, FYP30, and FYP40 in comparison to FYP10, a consistent decline in $\Delta$A was observed. The absorbance changes for these conditions were 0.82, 0.629, and 0.441, respectively. These diminishing values suggest a reduction in cell density with increasing fructose concentrations, indicating a potential inhibitory effect on cell growth or a shift in the strain’s preference for fructose concentration. It’s worth noting that there were no statistically significant differences between FYP20 and FYP, but FYP30 and FYP40 exhibited significantly lower $\Delta$A values compared to FYP, indicating a notable decrease in cell density under these higher fructose concentrations (Table 3, Fig. 7).

In summary, the $\Delta$A results indicate distinct growth patterns for the *F. tropaeoli* KKP 3032 strain under varying fructose concentrations. Particularly noteworthy is the remarkable increase in cell density observed in FYP10, exceeding that of FYP, suggesting a favorable condition for growth at 10% fructose. Despite the consistent decline in $\Delta$A as fructose concentration increased in FYP20, FYP30, and FYP40, highlighting a potential inhibitory effect or a shift in the strain’s fructose preference, it is crucial to acknowledge that even on FYP40, characterized by the highest fructose concentration (40%), there was still observable growth. This resilience indicates that the *F. tropaeoli* KKP 3032 strain possesses the capability to grow under conditions with high sugar concentrations, albeit at a reduced rate. These findings underscore the adaptability of the strain to varying fructose levels and its ability to sustain growth even in challenging environments.

There are bacteria known for their tolerance to high sugar concentrations. Notable examples include certain strains of *Leuconostoc mesenteroides*, which can tolerate sucrose concentrations of up to 60% (Huang et al. 2021). In a study by Behare et al. (2020), the tolerance of LAB strains to fructose was investigated. The highest survival rates were observed for the strain *L. mesenteroides* DPC 7261, with approximately 80% survival at 40% fructose and about 55% survival at 50% fructose.

Researchers also examined four strains of *Fructobacillus fructosus*, which displayed good but varied tolerance to fructose. The strains *F. fructosus* DPC 7235, DPC 7237, and DPC 7266 had survival rates of about 50%, 70%, and 66% respectively at 40% fructose, while their survival rates at 50% fructose were approximately 40%, 50%, and 50%. An interesting case was the strain *F. fructosus* DPC 7267, which showed a survival rate of about 75% at 40% fructose, but its survival drastically dropped to below 25% at 50% fructose. Additionally noteworthy is the strain *F. durionis* DPC 7238, which exhibited one of the highest survival rates at 40% fructose, exceeding 80% (Behare et al. 2020). Given the exceptional osmotolerance demonstrated by the strain KKP 3032, its remarkable growth in the presence of 40% fructose is noteworthy and atypical for the species *F. tropaeoli* (Endo and Okada 2008; Endo and Dicks 2014). This unusual characteristic could be leveraged in the food industry, particularly in the production of high-sugar foods, where microbiological stability is crucial.

### Probiotic potential

#### Resistance to low pH

To survive the journey through the human gastrointestinal tract and effectively exert their physiological effects, probiotics must be capable of enduring the challenging conditions of gastric juices and bile salts. Resistance to acidity and tolerance to bile are presently considered as primary screening criteria for potential probiotic strains.

The first challenge that probiotic bacteria must overcome is the low pH in the stomach, which is caused by the secretion of hydrochloric acid by mucosal cells. These unfavorable conditions serve as the initial protective barrier against foodborne pathogens while not interfering with the digestive processes in the stomach, where enzymes operate optimally (Olszewska and Łaniewska-Trokenheim 2013). The pH of the human stomach can reach levels as low as 1.0$-$1.5 (on an empty stomach). However, the ability to buffer the food matrix plays a significant role in determining the survival of ingested bacteria in the stomach. Therefore, in most in vitro tests, a pH of 3 is recommended (Zielińska et al. 2015; Patil et al. 2020).

**Fig. 8:**
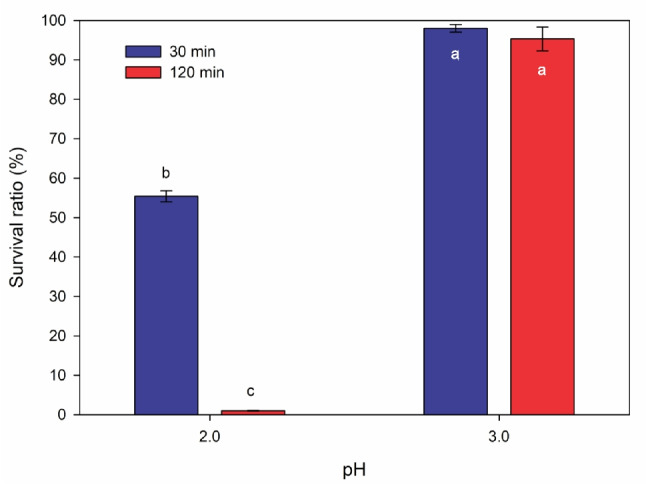
Resistance of *Fructobacillus tropaeoli* KKP 3032 to low pH of the environment (*n* = 3). Statistically significant differences between groups are indicated by different letters

The results of the low pH resistance tests revealed that *F. tropaeoli* KKP 3032 exhibited no tolerance to pH 1.5; the strain did not survive even during a 30-min incubation under these conditions. Incubating the KKP 3032 strain in an environment with a pH of 2.0 demonstrated a viability rate of 56% after 30 min of exposure, but it did not maintain its viability after 120 min of incubation. The survival ratio of the KKP 3032 strain following incubation in an environment with a pH of 3.0 did not change significantly and remained at a high level of approximately 98% and 95% after 30 min and 120 min of incubation, respectively. The influence of low pH on the viability of the *F. tropaeoli* KKP 3032 strain is depicted in Fig. 8.

An examination of the findings in this study indicates that the strain under investigation can persist in an acidic environment with a pH of 3.0 while retaining its vitality, thus implying its ability to survive under gastric conditions.

The tolerance to gastric acid is a fundamental property of every probiotic strain, crucial for its survival during the passage through the stomach (Patil et al. 2020). It is important to note that lactic acid bacteria, as they ferment sugars into lactic acid, continuously activate protective mechanisms against the harmful effects of the produced acid throughout their life cycle. The most significant roles are attributed to proton pumps like F1F0-ATPase and systems such as arginine deiminase (ADI), glutamate decarboxylase (GAD), and the ability to produce chaperones (Olszewska and Łaniewska-Trokenheim 2013; Wang et al. 2018). The production of ammonia (ADI system) can also significantly contribute to increased cell tolerance to low pH. It combines with protons present in the cytoplasm, forming NH_4_^+^ and thereby elevating intracellular pH (Meradji et al. 2023; Olszewska and Łaniewska-Trokenheim 2013).

The experimental conditions utilized in this study aimed to simulate the human gastric juice environment. Although not entirely representative, this approach is commonly adopted by many researchers for the rapid evaluation of potential probiotic properties of bacteria (Zielińska et al. 2015).

Other researchers achieved comparable results, observing that the *F. tropaeoli* TR581701 strain does not endure exposure to pH 1.0 and pH 2.0, but it does survive in an environment with a pH of 3.0. However, the survival rate of the TR581701 strain was considerably lower in comparison to the *F. tropaeoli* KKP 3032 strain, at around 60% (Simsek et al. 2022). Recently, an intriguing study conducted by a team of researchers, including the authors Patil et al. (2020), assessed another fructophilic strain, *F. fructosus* MCC 3996, with regard to its viability under both acidic pH and synthetic gastric juice conditions. The *F. fructosus* MCC 3996 strain maintained its vitality at levels of 67–68% at pH 2.0 and 3.0, whereas the survival rate in synthetic gastric juice (pH 2.0) reached 81%. The higher survival rate in synthetic gastric juice compared to low pH is likely due to the presence of additional dissolved substances, such as dextrose and metal ions. The strain did not survive exposure to pH 1.0.

#### Resistance to bile salts

Another barrier that probiotic bacteria must overcome is bile salts, which are produced in the liver from cholesterol and secreted into the duodenum to emulsify lipids. Studies evaluating the probiotic potential of bacteria require a thorough analysis of their survivability under varying concentrations of bile salts (Olszewska and Łaniewska-Trokenheim 2013). The physiological concentration of bile salts in the human gastrointestinal tract is variable, and different concentration ranges are reported depending on the source. While most authors suggest concentrations between 0.3 and 0.5% as typical levels of bile salts, some recommend using higher concentrations in simulations of the small intestine (Zielińska et al. 2015; Olszewska and Łaniewska-Trokenheim 2013; Niu et al. 2019; Ren et al. 2014). Consequently, in vitro survival tests were conducted at four different bile salt concentrations, covering all suggested ranges.

The *F. tropaeoli* KKP 3032 strain displayed remarkable tolerance to bile salts and maintained viability across all tested concentrations. The strain exhibited growth potential at concentrations of 0.3% and 0.5% bile salts, with survival rates of approximately 110.63% and 110.57% after 24 h of incubation, and 110.66% and 109.90% after 48 h of incubation, respectively. Additionally, strain KKP 3032 exhibited tolerance to bile salts at a concentration of 0.7%, with survival rates of 108.59% after 24 h and 109.35% after 48 h of incubation. Moreover, the strain demonstrated its survival capability in the presence of 1.5% bile salts, with survival rates exceeding 95.97% after 24 h and 95.38% after 48 h of exposure.

**Fig. 9:**
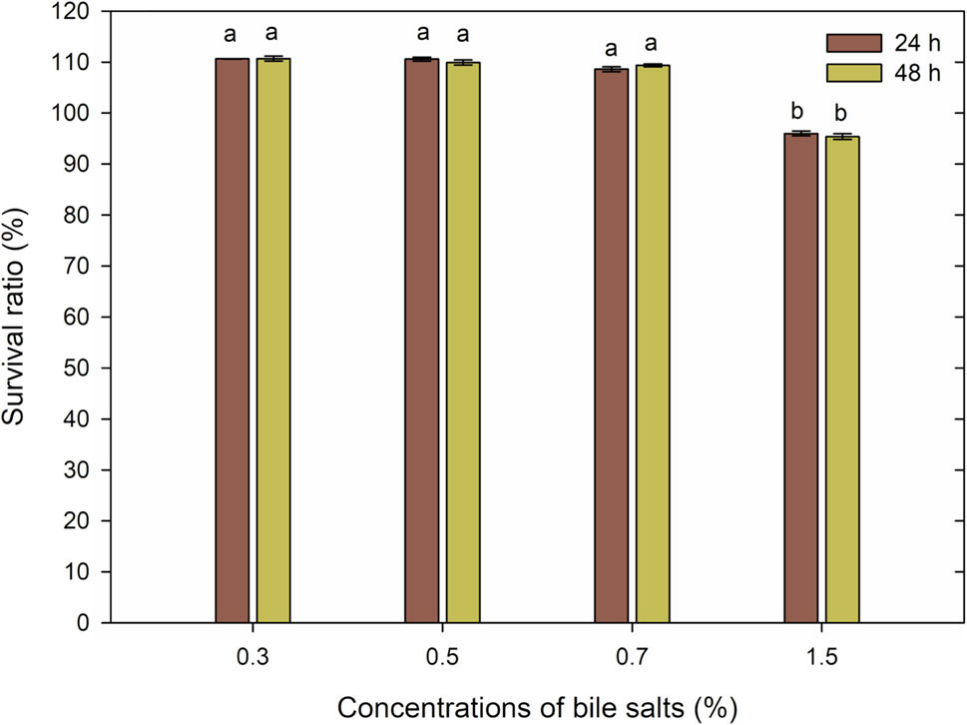
Resistance of *Fructobacillus tropaeoli* KKP 3032 strain to high percentage concentrations of bile salts. Initial count of bacteria was 7.02 log CFU/mL (*n* = 3). Statistically significant differences between groups are indicated by different letters

The results of our research show that the *F. tropaeoli* KKP 3032 strain can endure in an environment with a high concentration of bile salts while retaining its vitality, indicating its capacity to thrive in the small intestine. Following incubation with bile salts, a significant number of live cells persist. Detailed data regarding the tolerance of the *F. tropaeoli* KKP 3032 strain to bile salts are presented in Fig. 9.

In a study conducted by Sakandar et al. (2019), FLAB strains, including *F. durionis* JNGBKS5, *F. fructosus* JNGBKS2, and *F. pseudoficulneus* JNGBKS3, demonstrated survival of 52.50%, 64.20%, and 71.90%, respectively, when incubated with 0.4% bile salts. In contrast, Simsek et al. (2022) reported that *L. kunkeei* and *F. tropaeoli* strains exhibited moderate tolerance to bile salts. These strains displayed robust survival rates at 0.3% and 0.5% bile salt concentrations but did not exhibit tolerance to 1% bile salts.

Assessing bile tolerance is crucial when selecting potential probiotics because it has been demonstrated that strains with the highest bile tolerance strongly contribute to alleviating lactose intolerance symptoms (Ren et al. 2014).

#### Autoaggregation test

The ability to autoaggregate (the aggregation of bacterial cells of the same strain) and colonize the host’s intestinal mucosa is one of the most desirable characteristics of probiotic bacteria (Patil et al. 2020; Piekarska-Radzik and Klewicka 2020). Adhering to the intestinal epithelium is one of the fundamental mechanisms through which probiotic bacteria exert a beneficial influence on the host’s health, including strengthening the intestinal epithelial barrier, preventing pathogen colonization, and stimulating the host’s immune system (Niu et al. 2019; Rodak 2011).

In recent years, several researchers have linked adhesive properties to the intestinal lining with autoaggregation and the surface hydrophobicity of bacterial cells (Niu et al. 2019). Consequently, autoaggregation and surface hydrophobicity of *Fructobacillus tropaeoli* KKP 3032 strain cells were assessed in this study.

**Fig. 10:**
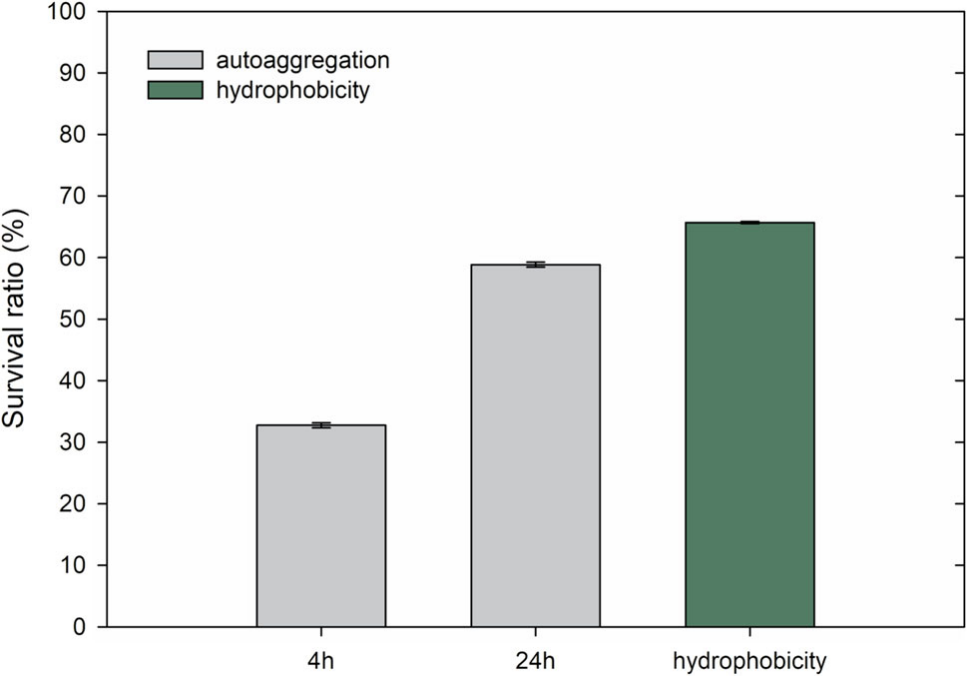
Resistance of *Fructobacillus tropaeoli* KKP 3032 strain to high percentage concentrations of bile salts. Initial count of bacteria was 7.02 log CFU/mL (*n* = 3)

The level of autoaggregation in *F. tropaeoli* KKP 3032 revealed that the highest percentage of autoaggregation was detected after 24 h, reaching 58.84%. This result indicates good autoaggregative properties of the *F. tropaeoli* KKP 3032 strain. In a similar study, Patil et al. (2020) observed that the *F. fructosus* strain exhibited autoaggregation at a level of 44.64% after 24 h. The researchers also noted that the degree of adhesion depended on several digestive enzymes, such as pepsin, trypsin, and bacterial protease. These enzymes reduced the level of autoaggregation by approximately 10%. Additionally, our results revealed that the level of autoaggregation gradually increased with prolonged incubation time (Fig. 10). Similar findings were reported by Dias et al. (2013), who observed an improvement in the autoaggregation abilities of *L. plantarum* strains as the incubation time increased. Detailed data regarding the autoaggregation of the *F. tropaeoli* KKP 3032 strain are presented in Fig. 10.

On the other hand, other researchers have observed significant variation in autoaggregation levels among *F. fructocus* isolates, ranging from approximately 25% to around 75%. Such divergent levels of autoaggregation within a single species are likely attributed to differences in the composition of surface molecules (proteins, EPS) among strains. These studies support the hypothesis that disparities observed in autoaggregation levels, even within the same species, are strain-specific and contingent upon bacterial surface molecules (Meradji et al. 2023).

Autoaggregation is an important property of *Fructobacillus tropaeoli* that may contribute to their effectiveness as potential probiotics. By forming aggregates, these bacteria can more effectively colonize the gastrointestinal tract, compete with pathogens. Although *Fructobacillus tropaeoli* is not yet used as a probiotic, its ability to autoaggregate makes it an interesting research subject for the development of new probiotic products.

**Table 4:** Antimicrobial activity of cell-free culture supernatants of *Fructobacillus tropaeoli* KKP 3032 against foodborne pathogenic bacteria (*n* = 3)

	Growth inhibition	Type of inhibition
Strain	Zone diameter [mm]	Zone*
*Listeria monocytogenes*	$26.30 \pm 0.47$	+++
KKP 1058		
*Escherichia coli* KKP 987	$25.70 \pm 0.47$	+++
*Pseudomonas aeruginosa*	$20.00 \pm 0.82$	++
KKP 994		
*Staphylococcus aureus*	$20.00 \pm 0.82$	++
KKP 995		
*Salmonella enterica* KKP 1044	$24.00 \pm 0.82$	+++
*Bacillus cereus* KKP 358	$15.70 \pm 0.47$	+

**Note:** Interpretation of zone inhibition diameter: + 11–16 mm; ++ 17–22 mm; +++ more than 23 mm

#### Cell surface hydrophobicity

The surface hydrophobicity of *Fructobacillus tropaeoli* KKP 3032 cells was quantified through photometric analysis employing the BATH assay with xylene. In this study, strain KKP 3032 exhibited approximately 65.69% hydrophobicity towards xylene (Fig. 10). According to previous reports, the minimum hydrophobicity value for a probiotic strain should be 40% (Niu et al. 2019). This result indicates a high affinity for xylene, an apolar solvent, confirming the hydrophobic nature of the strain’s surface. Our study results suggest that *F. tropaeoli* may colonize the surface of the intestinal mucosa, which exhibits a hydrophobic character due to the presence of these surface-active phospholipids (Patil et al. 2020).

**Table 5:** Minimal inhibitory concentration (MIC) of selected antibiotics in *Fructobacillus tropaeoli* KKP 3032 strain

Bacteria	AM	GM	KM	SM	EM	CM	TET	CL
*F. tropaeoli*	0.25	12	$>256$	64	0.75	0.047	1.5	1.5
KKP 3032								
EFSA standards*	2	16	64	64	1	4	8	4

**Note:** For obligate heterofermentative lactic acid bacteria

*AM* ampicillin, *GM* gentamycin, *KM* kanamycin, *SM* streptomycin, *EM* erythromycin, *CM* clindamycin, *TC* tetracycline, *CL* chloramphenicol

As mentioned previously, numerous researchers posit that a high surface hydrophobicity of cells can facilitate mucosal colonization and play a significant role in bacterial adhesion to epithelial cells (Zielińska et al. 2015). Conversely, Mathara et al. (2008) disagree with this assertion, as they did not uncover definitive evidence supporting a correlation between hydrophobicity and adhesion to epithelial cells. Their observations revealed that strains of *Lactobacillus* bacteria isolated from the environment, despite possessing high hydrophobicity, did not manifest adhesion capabilities or did so at a minimal level. Conversely, they encountered strains characterized by low hydrophobicity that exhibited robust adhesion to epithelial cells. Other authors, such as Lim and Ahn, contend that pronounced hydrophobicity in fermenting bacteria may indicate potential probiotic attributes in the studied strains. Nevertheless, they also propose that the adhesion of probiotic bacterial cells may hinge on environmental factors, encompassing medium composition, pH, temperature, and the presence of Ca^2+^ ions (Piekarska-Radzik and Klewicka 2020; Lim and Ahn 2012). Thus, while hydrophobicity may bolster adhesion, it does not constitute a mandatory prerequisite for robust adhesion (Zielińska et al. 2015).

#### Antimicrobial activity

The production of antibacterial compounds by lactic acid bacteria is an exceptionally vital trait among probiotics. It enables them to inhibit the growth of pathogens, compete with them, or even kill them (Sakandar et al. 2019). The antibacterial activity of *F. tropaeoli* KKP 3032 was assessed against several pathogenic bacteria, including *Listeria monocytogenes* KKP 1058, *Escherichia coli* KKP 987, *Pseudomonas aeruginosa* KKP 994, *Staphylococcus aureus* KKP 995, *Salmonella enterica* KKP 1044, and *Bacillus cereus* KKP 358. Strain *F. tropaeoli* KKP 3032 exhibited antibacterial activity against all tested foodborne pathogens. The highest antibacterial activity was observed against *Listeria monocytogenes* KKP 1068 (inhibition zone = 26.3 mm), while the lowest was against *Bacillus cereus* KKP 358 (15.7 mm). Detailed results of the antibacterial activity of strain *F. tropaeoli* KKP 3032 are presented in Table 4.

The antimicrobial activity of lactic acid bacteria is attributed to various bacterial metabolites, especially bacteriocins. In this study, the profile of compounds responsible for the antimicrobial activity of *F. tropaeoli* KKP 3032 was not investigated. However, it is presumed that organic acids, primarily, are the most active compounds involved in antagonistic mechanisms. De Simone et al. (2023) conducted research to determine the nature of compounds responsible for the antagonistic activity of *F. fructosus* AREP6. The results showed that, in addition to lactic acid, other organic acids such as acetic, citric, malic, tartaric, and succinic acids were involved in inhibiting the growth of pathogens.

Further research is needed to uncover the mechanisms underlying the antibacterial properties, including a detailed analysis of the metabolites produced by *F. tropaeoli* KKP 3032.

#### Safety evaluation — antibiotic susceptibility

One of the primary concerns regarding probiotic candidates pertains to evaluating their antibiotic resistance. Bacterial antibiotic resistance currently poses a significant issue. The widespread, excessive, and often improper use of antibiotics has led to a surge in the number of microorganisms resistant to various classes of antibiotics (Rodak 2011). Lactic acid bacteria have been acknowledged as safe and have received the GRAS (Generally Recognized as Safe) and QPS (Qualified Presumption of Safety) statuses from the FDA and EFSA. Nonetheless, recent research has unveiled that both among strains encompassed in starter cultures or probiotics, antibiotic-resistant strains can be identified as well. If resistance is an inherent (natural) trait, associated with genetic information encoded in the chromosome, there is no cause for alarm, as the likelihood of transferring resistance to another microorganism is minimal. Unfortunately, strains with acquired resistance, stemming from point mutations or gene transfer, are increasingly prevalent. Both of these phenomena ultimately result in the enduring inheritance of resistance and its dissemination via horizontal gene transfer (HGT). Additionally, disconcerting reports have emerged, indicating that LAB, including probiotic strains, are developing acquired resistance to an expanding array of antibiotic classes, particularly tetracyclines and macrolides (Chajecka-Wierzchowska and Zadernowska 2019). Other studies examining the antibiotic resistance of lactic acid bacteria have disclosed that the majority of them inherently resist aminoglycosides (Niu et al. 2019; Danielsen and Wind 2003). To thwart the undesirable transfer of resistance from endogenous microflora, probiotics should not exhibit acquired resistance (Niu et al. 2019).

The antibiotic resistance profile of the *F. tropaeoli* KKP 3032 strain was consistent with the recommendations provided by the European Food Safety Authority (EFSA) (Rychen et al. 2018), except for kanamycin, to which the strain exhibited resistance (Table 5). Kanamycin belongs to the aminoglycoside class of antibiotics, and according to current knowledge, resistance to this type of antibiotic does not pose a threat and is likely the result of inherent resistance (Niu et al. 2019). As mentioned earlier, the natural resistance of LAB and probiotics to antibiotics, in and of itself, is not hazardous; on the contrary, it can be a desirable trait for restoring gut microbiota during antibiotic therapy (Niu et al. 2019; Chajecka-Wierzchowska and Zadernowska 2019). Therefore, the primary factor determining the safety of a particular strain is the potential for resistance transfer rather than the mere presence of resistance. It is essential to determine whether resistance genes are located on mobile genetic elements or if they are an inherent trait (Chajecka-Wierzchowska and Zadernowska 2019).

Furthermore, a recently published study by Campedelli et al. (2018). in which the authors determined antibiotic susceptibility profiles for 182 strains of the *Lactobacillus* genus, revealed that many species exceed the antibiotic resistance levels recommended by EFSA. The authors suggested that the MIC values should be reevaluated, providing evidence for the need to revise regulatory guidelines regarding the safety assessment of probiotic strains.

The above-mentioned antibiotic resistance profile of *F. tropaeoli* KKP 3032 suggests that the strain most likely qualifies as a probiotic strain. However, future research should focus on the precise location and the potential for the transfer of kanamycin-resistance genes.

## Conclusions

The FLAB bacteria, particularly *Fructobacillus* spp., remain relatively understudied. However, due to their unconventional origins and specific growth preferences, they emerge as highly promising candidates for potential application across diverse industrial sectors. Consequently, in our pursuit to expand our understanding of these bacteria and discern their potential applications, we conducted a preliminary assessment of the probiotic potential and growth characteristics of the *F. tropaeoli* KKP 3032 strain. The comprehensive investigation into the growth dynamics, metabolic characteristics, and probiotic potential of *Fructobacillus tropaeoli* KKP 3032 has yielded valuable insights into its unique features.

The strain displayed distinctive growth patterns, demonstrating a preference for fructose-containing media, particularly under aerobic conditions. These observed growth characteristics align with the criteria established for fructophilic strains, suggesting that *F. tropaeoli* KKP 3032 thrives in environments rich in fructose, with a predilection for aerobic conditions. Intriguingly, the strain demonstrated remarkable adaptability to varying fructose concentrations, exhibiting optimal growth at 10% fructose. This adaptability, combined with exceptional osmotolerance, underscores the strain’s resilience in challenging environments, even when exposed to high sugar concentrations. Its capacity to endure acidic conditions and varying concentrations of bile salts further emphasizes its potential as a probiotic candidate capable of surviving the gastrointestinal tract, however, more detailed studies are necessary to confirm these properties.

Additionally, the autoaggregation and surface hydrophobicity of *F. tropaeoli* KKP 3032 suggest favorable adhesive properties, pointing to its potential to colonize the host’s intestinal mucosa. The strain displayed substantial hydrophobicity, a characteristic associated with mucosal colonization. Moreover, autoaggregation increased with prolonged incubation, indicating the strain’s ability to form aggregates, a desirable trait for probiotic adherence. The strain exhibited notable antibacterial activity, particularly against *Listeria monocytogenes* and *Salmonella enterica*, showcasing its ability to inhibit the growth of pathogenic bacteria.

The antibiotic resistance profile of *F. tropaeoli* KKP 3032 aligns with EFSA standards; however, the identified resistance to kanamycin prompts a more thorough investigation into the specific location and transfer potential of resistance genes. Acquiring precise knowledge regarding the location of these resistance genes is crucial for further stages of research. In summary, the confirmed preliminary probiotic potential of the *F. tropaeoli* strain, combined with its unique fructophilic trait, confirms the uniqueness of the strain under investigation.

Given the exceptional osmotolerance demonstrated by the strain KKP 3032, its remarkable growth in the presence of 40% fructose is noteworthy and atypical for the species *F. tropaeoli*. This unusual characteristic could be leveraged in the food industry, particularly in the production of high-sugar foods, where microbiological stability is crucial. Potential applications include using the KKP 3032 strain as a protective culture in fructose-rich products. Due to its ability to tolerate high fructose concentrations, KKP 3032 can limit the growth of undesirable microorganisms that are not adapted to such conditions. This combination can significantly extend product shelf life, minimizing the risk of microbiological spoilage and quality loss. Moreover, this strain can help maintain the desired organoleptic properties of products, such as taste and texture.

Additionally, this strain could be utilized to produce new fermented food products based on fructose-rich raw materials. Examples might include fermented fruit beverages, which would not only develop unique flavor profiles but also benefit from increased shelf life due to the efficient breakdown of fructose by KKP 3032. Furthermore, if the probiotic properties are confirmed, such products would contain beneficial bacteria. Such innovations could attract consumers seeking natural and durable food products.

The results of our study represent some of the first analyses suggesting that a strain belonging to the *F. tropaeoli* species could find application as a fructophilic probiotic or protective culture in fructose-rich foods, provided its probiotic potential is confirmed in *in vivo* studies. The findings of this study represent a substantial contribution to our comprehension of these distinctive bacteria, recognized for their noteworthy characteristics.

## Data Availability

All data will be made available on request.
