# Correction to: Characteristics and in vitro properties of potential probiotic strain *Fructobacillus tropaeoli* KKP 3032 isolated from orange juice

**DOI:** 10.1007/s12223-024-01233-5

**Published:** 2024-12-10

**Authors:** Anna Mikołajczuk-Szczyrba, Adrian Wojtczak, Marek Kieliszek, Barbara Sokołowska

**Affiliations:** 1 Department of Microbiology, Prof. Waclaw Dabrowski Institute of Agricultural and Food Biotechnology - State Research Institute, Rakowiecka 36 Street, Warsaw 02-532 Masovian Voivodeship, Poland; 2 Department of Food Biotechnology and Microbiology, Institute of Food Sciences, Warsaw University of Life Sciences—SGGW, Nowoursynowska 159C, 02-776 Warsaw, Poland

**Correction to: Folia Microbiologica**

10.1007/s12223-024-01207-7

The original version of the article unfortunately contained an error.

The affiliation for Marek Kieliszek should be corrected from “Microbiological Quality Research Laboratory, Department of Microbiology, Prof. Waclaw Dabrowski Institute of Agricultural and Food Biotechnology—State Research Institute, Rakowiecka 36 Street, Warsaw 02–532, Masovian Voivodeship, Poland” to “Department of Food Biotechnology and Microbiology, Institute of Food Sciences, Warsaw University of Life Sciences—SGGW, Nowoursynowska 159C, 02–776 Warsaw, Poland”.

The original article has been corrected.

